# Temporal and Racial Differences Associated with Atopic Dermatitis *Staphylococcus* *aureus* and Encoded Virulence Factors

**DOI:** 10.1128/mSphere.00295-16

**Published:** 2016-12-07

**Authors:** Joseph A. Merriman, Elizabeth A. Mueller, Michael P. Cahill, Lisa A. Beck, Amy S. Paller, Jon M. Hanifin, Peck Y. Ong, Lynda Schneider, Denise C. Babineau, Gloria David, Alexandre Lockhart, Keli Artis, Donald Y. M. Leung, Patrick M. Schlievert

**Affiliations:** a Department of Microbiology, University of Iowa, Iowa City, Iowa, USA; b National Jewish Health, Denver, Colorado, USA; c University of Rochester Medical Center, Rochester, New York, USA; d Northwestern University Feinberg School of Medicine, Chicago, Illinois, USA; e Oregon Health & Science University, Portland, Oregon, USA; f Keck School of Medicine of USC, Los Angeles, California, USA; g Harvard Medical School/Boston Children’s Hospital, Boston, Massachusetts, USA; h Rho, Chapel Hill, North Carolina, USA; University of Rochester

**Keywords:** atopic dermatitis, clonal groups, phenotype, race, *Staphylococcus aureus*, superantigens

## Abstract

Atopic dermatitis (AD) is an inflammatory skin condition strongly associated with *Staphylococcus aureus* colonization and infection. *S. aureus* strains shift in populations in ~10-year intervals depending on virulence factors. Shifts in *S. aureus* virulence factors may in part explain the racial differences observed in the levels of prevalence and severity of AD. AD *S. aureus* isolates collected from 2011 to 2014 (103 isolates) and in 2008 (100 isolates) were examined for the prevalence of genes encoding superantigens (SAgs). The strains from 2011 to 2014 were obtained from AD patients as a part of the National Institute of Allergy and Infectious Diseases (NIAID) Atopic Dermatitis Research Network (ADRN). The prevalence of SAg genes was investigated temporally and racially. The enterotoxin gene cluster (EGC) was more prevalent in the 2011–2014 AD isolates than in the 2008 AD isolates. The prevalences of virulence factor genes were similar in European American (EA) and Mexican American (MA) patients but differed in 6 of 22 SAg genes between EA and African American (AA) or MA and AA isolates; notably, AA isolates lacked *tstH*, the gene encoding toxic shock syndrome toxin 1 (TSST-1). The presence of *tstH* and *sel-p* (enterotoxin-like P) was associated with decreased clinical severity and increased blood eosinophils, respectively. The EGC is becoming more prevalent, consistent with the previously observed 10 years of cycling of *S. aureus* strains. Race-specific *S. aureus* selection may account for differences in virulence factor profiles. The lack of TSST-1-positive (TSST-1^+^) AD *S. aureus* in AA is consistent with the lack of AAs acquiring TSST-1-associated menstrual toxic shock syndrome (TSS).

**IMPORTANCE** Monitoring pathogen emergence provides insight into how pathogens adapt in the human population. Secreted virulence factors, important contributors to infections, may differ in a manner dependent on the strain and host. Temporal changes of *Staphylococcus aureus* toxigenic potential, for example, in encoding toxic shock syndrome toxin 1 (TSST-1), contributed to an epidemic of TSS with significant health impact. This study monitored changes in atopic dermatitis (AD) *S. aureus* isolates and demonstrated both temporal and host infection differences according to host race based on secreted superantigen potential. The current temporal increase in enterotoxin gene cluster superantigen prevalence and lack of the gene encoding TSST-1 in AAs predict differences in infection types and presentations.

## Abstract (precis)

Monitoring pathogen emergence provides insight into how pathogens adapt in the human population. Secreted virulence factors, important contributors to infections, may differ in a manner dependent on the strain and host. Temporal changes of *Staphylococcus aureus* toxigenic potential, for example, in encoding toxic shock syndrome toxin 1 (TSST-1), contributed to an epidemic of TSS with significant health impact. This study monitored changes in atopic dermatitis (AD) *S. aureus* isolates and demonstrated both temporal and host infection differences according to host race based on secreted superantigen potential. The current temporal increase in enterotoxin gene cluster superantigen prevalence and lack of the gene encoding TSST-1 in AAs predict differences in infection types and presentations.

## INTRODUCTION

Atopic dermatitis (AD) is the most common chronic inflammatory skin disease in the general population. AD is often a T helper 2 (Th2)-mediated disease, accompanied by increased serum total IgE production, circulating interleukin-4 (IL-4)/IL-13-expressing T cells, and eosinophilia (1–3). It is often associated with skin infections caused by herpes simplex virus and *Staphylococcus aureus* (4).

*S. aureus* is capable of producing a myriad of virulence factors, allowing it to be a multidimensional pathogen. Sortase and covalently attached surface adhesin molecules confer colonization properties, cytolysins cause acute, localized keratinocyte toxicity and inflammation, and superantigens (SAgs) act locally and systemically to dysregulate the host immune response, thereby interfering with immunity (5).

Classically, SAgs function by cross-linking the variable part of the β-chain of the T-cell receptor (Vβ-TCR) and α and/or β chains of the major histocompatibility complex II (MHC II) molecules, leading to potent proinflammatory responses, sometimes termed cytokine storms (6). The nomenclature for staphylococcal SAgs indicates their primary disease associations. Staphylococcal enterotoxins (SEs) A to E and G cause emesis in humans and nonhuman primates. Toxic shock syndrome toxin 1 (TSST-1), differing in its primary amino acid sequence from other SAgs, is the cause of all cases of menstrual TSS (mTSS) and 50% of nonmenstrual cases. The remaining repertoire of SAgs related to SEs either lacks emetic activity or has not been tested, and thus they are labeled staphylococcal enterotoxin-like (SEl) molecules. The enterotoxin gene cluster (EGC) is composed of 6 superantigen genes, *seg*, *sel-i*, *sel-m*, *sel-n*, *sel-o*, and *sel-u*, that are increasingly recognized as important to staphylococcal disease (3), despite previously being considered a cluster or “nursery” of SAg genes of unknown function or potentially giving rise to new toxins through recombination (7).

Recently, SAgs have been shown to interact with and elicit proinflammatory responses from epithelial cells (8). This interaction leads to a local immunological response that can be followed by the systemic symptoms of TSS, through a mechanism known as outside-in signaling. Support for the idea of SAg roles in AD comes from a recent study characterizing the SAg profile of *S. aureus* isolated from AD patients who were resistant to steroid treatment, the most common anti-inflammatory therapy in AD, as well as from those who were not (9). Significant differences were seen in the numbers and types of SAgs encoded by the isolates infecting steroid-resistant patients compared to those encoded by isolates infecting steroid-sensitive patients, indicating that different *S. aureus* isolates were preferentially infecting those differing host environments.

As of 2003, the overall prevalence of AD in children was over 10% (10). Further investigation of host race depicts a significant difference in prevalence between African American (AA) and European American (EA) children, 15.9% and 9.7%, respectively (10). Multiple studies have attempted to correlate prevalences of AD as well as differences in disease severity with host race by examining differences in stratum corneum ceramide composition, transepithelial water loss (TEWL) (11), pH (12), filaggrin mutations (13), and nasal carriage of *S. aureus*. No one of these factors alone was responsible for variable AD presentation in AA versus EA patients, suggesting that other factors may be responsible for the observed differences. Interestingly, racial differences in immune activation have recently been identified in AD patients (14).

*S. aureus* can be found in 40 to 100% of AD lesions and at levels as high as 10^7^ CFU/cm^2^ (15). Antibiotic use leads to reduction of lesions, demonstrating that *S. aureus* infection functions critically in disease progression and persistence (16). Many known host factors in AD vary by host race, and yet no studies to date have classified *S. aureus* strains infecting patients of different racial backgrounds.

Through the identification of the SAg profile of lesional AD isolates, we aimed to discern differences in *S. aureus* strains between the 2008 (9) and 2011–2014 time periods, as well as to differentiate the strains that infect EA, AA, and Mexican American (MA) AD patients.

## RESULTS

### Enterotoxin gene cluster (EGC) genes were carried more frequently in the 2011–2014 lesional AD isolates than in the 2008 AD isolates.

*S. aureus* was isolated from lesions of 103 AD patients from 2011 to 2014. Age, sex, eczema area and severity index (EASI) score, total serum IgE level, and eosinophil count for AD patients providing isolates from 2011 to 2014 are summarized by host race in Table 1. Of the 103 AD patients, 50 were EA, 27 AA, and 26 MA. Other than being from AD patients from diverse geographic locations in the United States, no additional demographics were available for the 100 patients who provided isolates in 2008. However, the diversity in SAg gene profiles in those isolates supports the evaluation that the strains tested were not from a clonal outbreak of AD.

**TABLE 1:** Demographic characteristics of patients with sample collection from 2011 to 2014^a^

Parameter	Values		
	African Americans (*n* = 27)	European Americans (*n* = 50)	Mexican Americans (*n* = 26)
Age (yrs)	20 (8, 26)	13 (9, 37)	12 (6, 19)
Sex			
Female	17 (63)	24 (48)	11 (42)
Male	10 (37)	26 (52)	15 (58)
Total serum IgE (kU/liter)	946 (253, 2,358)	1,175 (261, 5,457)	955 (188, 2,721)
EASI score	13 (8, 22)	20 (7, 29)	20 (8, 29)
Eosinophil count (cell count/mm^3^)	422 (231, 625)	445 (147, 706)	420 (219, 690)

a All statistics are median (25th quartile, 75th quartile), except the sex data, which represent the number (percent) of patients.

For the 2011–2014 isolates, SAg genes associated with the EGC (*sel-i*, *seg*, *sel-m*, *sel-n*, and *sel-o*) were the most frequently represented SAg genes, with >50% of the isolates carrying one or more of the EGC genes (Table 2). *sel-u* is also carried by the EGC, but this SAg gene was not evaluated in the 2008 study and thus is not reported in the comparison in Table 2. However, among the 2011–2014 isolates (*n* = 46) that had all five of the other EGC genes, 100% also contained *sel-u*.

**TABLE 2:** Comparison of SAg prevalence levels over time

SAg gene	No. (%) of strains positive for indicated gene		*P* value^a^
	2008 AD (*n* = 100)	2011–2014 AD (*n* = 103)	
*sea*	41 (41)	7 (6.8)	<0.0001
*seb*	35 (35)	6 (5.8)	<0.0001
*sec*	25 (25)	11 (11)	0.009
*sed*	39 (39)	16 (16)	0.0002
*see*	39 (39)	0 (0)	<0.0001
*seg*	55 (55)	70 (68)	0.06
*sel-h*	43 (43)	2 (1.9)	<0.0001
*sel-i*	48 (48)	59 (57)	0.20
*sel-j*	69 (69)	13 (13)	<0.0001
*sel-k*	49 (49)	16 (16)	<0.0001
*sel-l*	29 (29)	5 (4.9)	<0.0001
*sel-m*	64 (64)	59 (57)	0.39
*sel-n*	51 (51)	71 (69)	0.009
*sel-o*	37 (37)	55 (53)	0.02
*sel-q*	40 (40)	12 (12)	<0.0001
*tstH*	38 (38)	10 (9.7)	<0.0001

a *P* values are based on comparisons of the prevalence levels of each SAg between the 2008 AD cohort and the 2011-to-2014 AD cohort.

A comparison of the representation of 16 SAg genes in 103 AD isolates from 2011 to 2014 to those in 100 AD isolates previously studied in 2008 (9) revealed a significant shift in the prevalence of selected SAg genes. Notably, SAg genes associated with the EGC (*sel-n* and *sel-o*) were significantly (*P* < 0.05) more prevalent in isolates from 2011 to 2014 than in those from 2008. In contrast, a significant (*P* < 0.05) reduction in the prevalences of *tstH* (the gene for TSST-1), *sel-q*, *sed*, *sel-j*, and *sea* was observed in the 2011–2014 isolates compared to the 2008 isolates (Table 2).

### SAg profiles of 2011–2014 AD isolates describe three genotypic groups.

**FIG 1:**
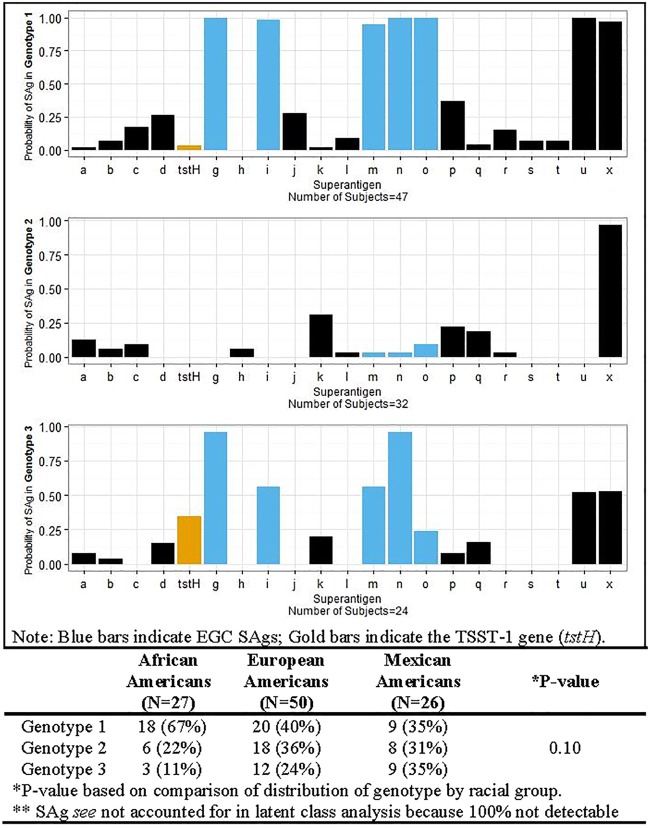
Probability of superantigen for a given *S. aureus* genotype. The table component of the figure displays the probability of each *S. aureus* genotype within each racial group.

*S. aureus* strains are commonly clonal grouped by overall chromosome arrangement, using, for example, pulsed-field gel electrophoresis. Alternatively, through the use of a latent class analysis, patients from 2011 to 2014 with similar SAg profiles were grouped into three major genotypes using 22 SAgs (Fig. 1). The basis for this analysis is that such studies allow groupings based on the virulence factors present versus chromosomal DNA patterns that may or may not reflect virulence trait differences. Genotype 1 included patients with strains that carried the EGC SAgs (*seg*, *sel-i*, *sel-m*, *sel-n*, *sel-o*, and *sel-u*) and *sel*-*x* ≥97% of the time. Genotype 1 also had a higher probability of carrying *sel-p* (enterotoxin-like P) (37%), *sel-j* (28%), *sed* (26%), *sec* (17%), *sel-r* (15%), *sel-l* (9%), *sel-s* (7%), and *sel-t* (7%) than the other two genotypes. Similarly to genotype 1, genotype 2 had a high probability of carrying *sel*-*x* (≥97%). Genotype 2 appears to be largely defined by only containing *sel-x*, suggesting that this group contains high numbers of USA300s (17) based on SAg profiles. Genotype 2 also had a ≥13% probability of carrying *sel-k*, *sel-p*, *sel-q*, and *sea* (Fig. 1). Genotype 3 had the highest (35%) probability of carrying *tstH* and had a ≥50% probability of encoding the majority of EGC SAgs and carrying *sel-x* (Fig. 1).

### SAg genotypes suggest differences as a function of host race.

Examination of the association between racial groups and *S. aureus* SAg profiles demonstrated that among AA isolates, 67% were in genotype 1, whereas EA and MA isolates were relatively evenly distributed across all three genotype groups (Fig. 1). Among AA isolates, 89% were in genotypes 1 and 2, which were those most notably lacking *tstH*. A comparison of the distributions of genotypes by race indicated no significant statistical differences, however.

### Pairwise comparison of individual SAg genes by host race indicates some SAg differences.

**FIG 2:**
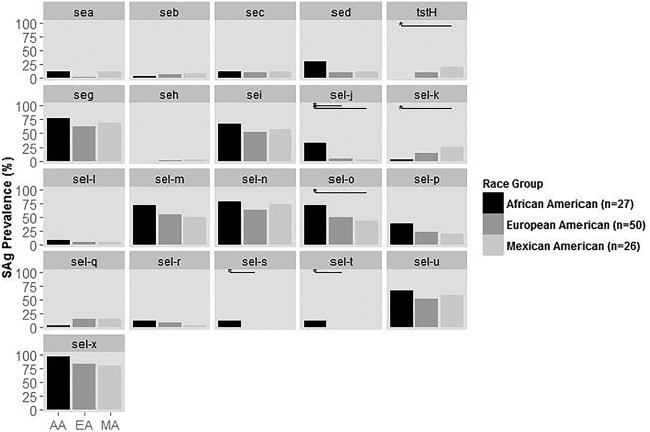
Prevalence of superantigen (SAg) genes in *S. aureus* isolates from 2011 to 2014 within each racial group. Horizontal lines indicate comparisons that are statistically significant at the 0.05 level.

Using the isolates collected from 2011 to 2014, a comparison of the prevalences of each SAg gene among racial groups demonstrates some significant differences (Fig. 2). There were no significant differences in the prevalences of any of the 21 SAg genes between EA and MA isolates (Fig. 2). Significant differences in the prevalences of 3 of 21 and 4 of 21 SAg genes were observed between EA and AA isolates and MA and AA isolates, respectively. MA isolates had significantly higher prevalences of *tstH and sel-k* and significantly lower prevalences of *sel-o* compared to AA isolates. EA and MA isolates lacked *sel-s* and *sel-t*, with AA isolates being the only strains containing these genes. AA isolates also more frequently carried *sel-j* and genes comprising the EGC than EA and MA isolates.

### EASI scores and eosinophil counts show an association with *tstH* and *sel-p*, respectively, but not host race.

There was no significant difference in EASI scores, total serum IgE levels, or eosinophil counts among the racial groups (data not shown). On the SAg gene level, isolates carrying *tstH* had a lower EASI score than isolates lacking the gene (mean difference, −9.3; 95% confidence interval [CI], −18.5, −0.2; *P* = 0.05) (Table 3, EASI score data). Isolates containing *sel-p* had significantly higher eosinophil counts than isolates lacking this gene (GMR, 1.6; 95% CI, 0.03, 2.4; *P* = 0.04) (Table 3, eosinophil count data).

**TABLE 3:** Adjusted mean differences in disease severity or geometric mean ratio in biomarkers (total serum IgE and eosinophils) between isolates with the given SAg gene and isolates without the given SAg gene^a^

SAg	EASI score		Total serum IgE (kU/liter)		Eosinophil count (cells/mm^3^)	
	Mean difference (95% CI)	*P* value	GMR (95% CI)	*P* value	GMR (95% CI)	*P* value
*sea*	−5.3 (−16.2, 5.7)	0.34	0.85 (0.18, 4.0)	0.83	0.70 (0.33, 1.5)	0.35
*seb*	6.4 (−5.5, 18.2)	0.29	0.57 (0.11, 3.0)	0.50	0.94 (0.42, 2.1)	0.89
*sec*	1.3 (−7.7, 10.3)	0.77	0.46 (0.13, 1.6)	0.22	0.90 (0.49, 1.7)	0.73
*sed*	1.7 (−6.0, 9.3)	0.67	2.3 (0.81, 6.8)	0.12	0.95 (0.56, 1.6)	0.84
*tstH*	−9.3 (−18.5, −0.2)	0.05*	0.74 (0.2, 2.8)	0.66	0.67 (0.36, 1.3)	0.21
*seg*	0.21 (−5.8, 6.3)	0.95	0.98 (0.42, 2.3)	0.97	0.76 (0.50, 1.1)	0.17
*sel-h*	−10.5 (−30.4, 5.3)	0.30	2.9 (0.18, 48.0)	0.45	1.2 (0.29, 4.4)	0.87
*sel-i*	1.5 (−4.2, 7.2)	0.60	0.88 (0.39, 2.0)	0.75	0.78 (0.53, 1.1)	0.19
*sel-j*	6.5 (−1.8, 14.8)	0.12	2.8 (0.84, 9.4)	0.09	1.2 (0.62, 2.0)	0.71
*sel-k*	−2.6 (−10.5, 5.3)	0.51	1.7 (0.58, 5.3)	0.32	1.5 (0.86, 2.5)	0.15
*sel-l*	−4.6 (−17.5, 8.2)	0.48	0.87 (0.14, 5.3)	0.88	0.71 (0.30, 1.7)	0.44
*sel-m*	2.6 (−3.0, 8.3)	0.36	1.2 (0.56, 2.8)	0.59	1.1 (0.72, 1.6)	0.75
*sel-n*	1.5 (−4.6, 7.6)	0.63	1.3 (0.56, 3.1)	0.54	0.87 (0.58, 1.3)	0.51
*sel-o*	3.4 (−2.1, 9.0)	0.22	1.6 (0.73, 3.5)	0.24	1.1 (0.72, 1.6)	0.75
*sel-p*	1.2 (−5.2, 7.6)	0.71	2.3 (0.93, 5.5)	0.07	1.6 (1.03, 2.4)	0.04*
*sel-q*	−0.60 (−9.5, 8.3)	0.89	1.4 (0.39, 4.7)	0.62	1.5 (0.82, 2.7)	0.18
*sel-r*	7.2 (−3.1, 17.5)	0.17	2.6 (0.62, 11.2)	0.19	1.79 (0.87, 3.5)	0.12
*sel-s*	10.9 (−5.7, 27.5)	0.19	1.7 (0.1, 30.0)	0.70	0.32 (0.08, 1.3)	0.10
*sel-t*	10.9 (−5.7, 27.5)	0.19	1.7 (0.1, 30.0)	0.70	0.32 (0.08, 1.3)	0.10
*sel-u*	1.3 (−4.3, 7.0)	0.64	0.94 (0.42, 2.1)	0.89	0.77 (0.52, 1.1)	0.17
*sel-x*	5.7 (−2.3, 13.7)	0.16	2.1 (0.7, 6.4)	0.20	1.3 (0.76, 2.3)	0.33

a All models adjusted for age at enrollment and sex. Statistically significant values are bolded and denoted with an asterisk (*). EASI, eczema area and severity index; GMR, geometric mean ratio.

### *S. aureus* from menstrual TSS isolates encoded TSST-1, but none of these were obtained from AA patients.

AA AD isolates of *S. aureus* lacked the *tstH* gene (Fig. 2). The Schlievert laboratory identified TSST-1 in 1981 (18) and has collected menstrual TSS isolates continually since then. Retrospective investigation of >6,000 menstrual TSS isolates revealed that no TSS isolate was from an AA patient.

## DISCUSSION

Treatment of the chronic inflammatory skin disease AD is costly; upward of a billion dollars per year in health care costs goes into managing symptoms associated with the disease. Further complexity in treatment stems from the fact that patients from different racial backgrounds respond differently to treatments (19). These differences are not associated with previously identified host barrier defects such as stratum corneum ceramide composition, TEWL, skin pH, or *S. aureus* nasal carriage (13, 20–23).

This study was undertaken to examine a possible temporal shift in SAg profiles over the past 3 to 6 years and to classify *S. aureus* isolates from AD patients from different racial backgrounds on the basis of virulence factor profile. The myriad of virulence factors encoded by *S. aureus* give it the ability to evade host immune elimination and additionally provide niche selection for colonization and infection. For example, patients who develop menstrual TSS do not develop immunity to their infecting *S. aureus* strain due to lack of protective response in the presence of TSST-1, and they remain susceptible to lifelong recurrences (24).

Analyses of AD isolates from 2011 to 2014 have shown a significantly higher prevalence of genes encoding EGC SAgs and a significantly lower prevalence of *tstH*, *sel-q*, and* sea* than analyses of AD isolates from 2008. We expected a shift in SAg profile over this time period, and our results engender particular concern with the emergence of the EGC. The EGC is a cluster of 6 SAgs and is intact in 2011–2014 isolates; this cluster was occasionally present in 2008, but in many strains collected in 2008 the cluster was broken up such that few strains carried all 6 EGC members. Results presented in this study suggest that in-depth patient information is important in analyzing the toxigenic potential of *S. aureus* isolates and that a lack of such information limits the utility of results obtained in comparing two pools of isolates. However, our data suggest that the toxigenic potential of strains changes over time and that such changes warrant further study. For example, important shifts in clonal groups have been correlated with the emergence of mTSS.

This shift to greater numbers of encoded EGC SAgs predicts that those SAgs should be more prevalent in disease isolates today. This is clearly the case for AD patients. However, significantly more *S. aureus* isolates from infective endocarditis, pneumonia, and bloodstream infections are also carrying the intact EGC. We have shown that SAgs contribute to development of AD and that SAgs are required for infective endocarditis, pneumonia, and bloodstream infections due to *S. aureus* (25–27). Furthermore, we have recently shown in a sensitive rabbit model (28) that EGC SAgs contribute to both pneumonia and infective endocarditis (3). It is noteworthy that the majority of infective endocarditis strains encode the EGC and that *S. aureus* has simultaneously become the leading cause of native valve infective endocarditis (29).

It is possible that the increased prevalence of the EGC results from development of immunity against a once-prevalent toxin, i.e., TSST-1. This would confer a positive selection of strains lacking this toxin, allowing them to become more prevalent. It is known that different *S. aureus* strains cycle through the human population in roughly 10-year intervals, and the changes seen in SAg genes may be a reflection of the cycling. For example, the 80/81 clonal group of *S. aureus* emerged in hospitals in about 1950, peaking to cause 85% of hospital infections in 1955 and disappearing by 1960. This clonal group was replaced in 1960 by the 52/52-A clonal group, which peaked in hospitals in 1965 and disappeared by 1970. Finally, this clonal group was replaced by clonal group USA200 (also known as 29/52), which produces TSST-1. The last-named clonal group also accounted for the emergence of TSST-1 in 1972 and for that seen in association with high-absorbency tampons in 1975 (30). Today, the incidence of menstrual TSS, which is caused by TSST-1 at a rate of nearly 100%, is lower than it was in the early 1980s, when the USA200 strain was emerging (31, 32).

Further analyses of the 2011–2014 isolates of AD showed that the isolates can be divided into three genotype groups, depending on their SAg profiles. We believe that these genotype groupings provide an emergent, important tool for epidemiological analysis of *S. aureus* associated with diseases. To date, the majority of clonal groupings of *S. aureus* have depended on chromosomal DNA analyses, for example, pulsed-field gel electrophoresis. This new genotype method studies the actual virulence factors required for human diseases. Genotype group 1 was characterized by carrying the EGC and SE*l-*X. Genotype group 3 has the highest probability of encoding TSST-1, among other SAgs. It is expected that genotype group 1 will continue to emerge epidemiologically and to be associated with a myriad of significant human diseases due to the presence of the EGC. Genotype group 3 strains would be expected to cause menstrual and nonmenstrual TSS due to the presence of the TSST-1 gene and should be associated with infective endocarditis, as has been seen, due to the presence in many strains of the EGC.

Examining genotype grouping based on host race revealed that ~90% of AA isolates were in genotype groups 1 and 2, which lacked the gene for TSST-1. The three genotype groups were relatively evenly distributed across MA and EA isolates. A trend in the prevalence among genotype groups based on host race may suggest that SAgs confer colonization or infection benefits that are dependent in part on host race. In contrast, host factors are likely to exert selective pressures against *S. aureus* producing TSST-1. Our observations of the lack of TSST-1-producing *S. aureus* strains in AAs merit further investigation, particularly of genetic differences among the host races that could explain these findings.

The total EASI scores are not significantly different across AAs, EAs, and MAs. However, patients colonized with strains containing *tstH* had a significant decrease in EASI score compared to patients colonized with isolates lacking this gene. Results from patients colonized with strains containing *sel*-*p* were significantly associated with increased eosinophil counts compared to those from patients colonized with isolates lacking this gene. Other SAg results approached statistical significance in correlation to increased EASI, IgE, or eosinophil counts. These data collectively suggest that the capacity of an infecting *S. aureus* strain to produce certain SAgs may contribute to disease severity (Table 3).

In sum, this study was the first of its kind to analyze the complete *S. aureus* SAg profile of AD patients across time and by racial background. Our data indicate that the EGC continues to emerge as the dominant SAg group, associated with many kinds of *S. aureus* infections, including AD. Our data presented here also suggest the *S. aureus* strains infecting AAs, EAs, and MAs have significant differences (Fig. 2). This may lead to differences in the prevalence and severity of diseases. The development of the SAg genotype method for clonal grouping is in its infancy, but we have already shown epidemiologically that the method can be used to analyze SAg distribution and associations. We expect its use to increase significantly, as it is increasingly recognized that SAgs contribute importantly to all or nearly all *S. aureus* infections.

## MATERIALS AND METHODS

### 2011–2014 patient enrollment and data collection.

Patients (*n* = 103) with AD were enrolled at six centers across the United States as part of the Atopic Dermatitis Research Network (ADRN) Registry study (ClinicalTrials.gov NCT01494142). Patients were administered a standardized set of questionnaires and underwent a physical examination (performed by a qualified medical professional) as well as blood and skin swab collection. The questionnaires, exam, and samples were used to evaluate their overall medical history and disease status. AD severity was assessed using the standardized eczema area and severity index (EASI) grading score, a sensitive clinical measure of the presentation and spread of inflamed skin at a single point in time. A higher EASI score (score range 0 to 72) equates to more-severe and extensive skin involvement. A complete blood count (CBC) with differential was performed on blood samples at Quest Diagnostics Laboratory. Total serum IgE (kU/liter) was measured by ImmunoCAP tests (Thermo Fisher Scientific) at the Johns Hopkins Asthma and Allergy Center (JHAAC). Patients were asked to self-identify their race and ethnicity from a list of 2010 United States Census categories. Patient age was calculated from self-reported date of birth to the date of study enrollment.

### Bacterial strains and PCR identification of SAgs.

Bacterial strains from 2011 to 2014 (*n* = 103) were isolated from swabs of lesional AD skin and were grown overnight in 25-ml Todd Hewitt broth cultures at 37°C and 200 revolutions/min. DNA samples from all 103 isolates from 2011 to 2014 were prepared using a Qiagen DNeasy blood and tissue kit. PCRs were carried out with *Taq* polymerase (Qiagen) along with known positive controls for all 22 known SAgs by using primers previously utilized (33). Positive DNA controls for PCR screening were obtained from laboratory strains maintained in a lyophilized state in the laboratory.

The data from a prior AD study published in 2008 were used for comparison to more recent isolates. Those prior isolates were lesional isolates from a general population of AD patients obtained in 2002 from diverse geographic locations. Since the prior 2008 AD study (9), six new SAgs have been described which were not assessed in the 2008 study. Therefore, analyses examining SAg profiles over time include only the 16 SAgs which were analyzed in common.

The *S. aureus* strains used in this study are maintained by and available as low-passage-number stocks through the NIH-funded Atopic Dermatitis Research Network (ADRN). The ADRN steering committee receives requests and approves release of samples to researchers, with authors Patrick M. Schlievert and Gloria David as contact individuals.

### Statistical methods.

Within each racial group, age data, total serum IgE levels, EASI scores, and eosinophil counts were summarized by medians and quartiles and sex was summarized using frequencies and percentages. Fisher’s exact tests were used to compare the prevalences of all SAgs between the 2011–2014 cohort and the 2008 cohort. A latent class analysis, performed via the poLCA package in R, was used to cluster isolates into SAg classes (or genotypes). The number of potential classes, ranging from 2 to 5, was examined using 500 iterations performed in each algorithm run. For each set of classes, the distribution of the model fit, measured by the Bayesian information criterion, was examined across each run. Three classes showed the least variability and best model fit across all runs. The prevalences of all SAgs were compared between racial groups using an unadjusted logistic regression model. Exact logistic regression was used to account for a low SAg prevalence of *tstH*, *sel-q*, *sel-s*, and *sel-t*. Multivariable generalized linear models adjusting for age and sex were used to compare individual outcomes of EASI scores, total serum IgE levels, and eosinophil counts between racial groups or between isolates with or without each SAg. No adjustments were made for multiple comparisons. All analyses were conducted using R software (version 3 to 2.2).
